# Positron spectroscopy of point defects in the skyrmion-lattice compound MnSi

**DOI:** 10.1038/srep29109

**Published:** 2016-07-08

**Authors:** Markus Reiner, Andreas Bauer, Michael Leitner, Thomas Gigl, Wolfgang Anwand, Maik Butterling, Andreas Wagner, Petra Kudejova, Christian Pfleiderer, Christoph Hugenschmidt

**Affiliations:** 1 Physik-Department, Technical University of Munich, 85748 Garching, Germany; 2 Heinz Maier-Leibnitz Zentrum, Technical University of Munich, 85748 Garching, Germany; 3 Institut für Strahlenphysik, Helmholtz-Zentrum Dresden-Rossendorf, 01314 Dresden, Germany

## Abstract

Outstanding crystalline perfection is a key requirement for the formation of new forms of electronic order in a vast number of widely different materials. Whereas excellent sample quality represents a standard claim in the literature, there are, quite generally, no reliable microscopic probes to establish the nature and concentration of lattice defects such as voids, dislocations and different species of point defects on the level relevant to the length and energy scales inherent to these new forms of order. Here we report an experimental study of the archetypical skyrmion-lattice compound MnSi, where we relate the characteristic types of point defects and their concentration to the magnetic properties by combining different types of positron spectroscopy with ab-initio calculations and bulk measurements. We find that Mn antisite disorder broadens the magnetic phase transitions and lowers their critical temperatures, whereas the skyrmion lattice phase forms for all samples studied underlining the robustness of this topologically non-trivial state. Taken together, this demonstrates the unprecedented sensitivity of positron spectroscopy in studies of new forms of electronic order.

Intermetallic compounds attract tremendous interest as a playground for novel electronic phases, such as unconventional superconductivity, partial forms of the electronic and spin order, emergent behaviour such as magnetic monopoles in the spin ices and frustration in spin liquids. In general, the preparation of high-quality single crystals of the materials of interest is extremely demanding, since a large number of mechanisms lead to the formation of intrinsic point defects. Examples include the formation due to configuration entropy, kinetically inhibited atomic motion during solidification and frozen-in disorder. The different species of defects comprise of, e.g., point defects such as vacancies and antisite disorder, dislocations or vacancy clusters. Despite their great relevance, the qualitative and quantitative identification of point defects has been very challenging technically. For instance, scanning tunneling microscopy is a surface sensitive probe only, whereas transmission electron microscopy introduces ambiguities, as the sample has to be machined thinned and prepared mechanically. Further, the degree of long-range order is conventionally accessible by diffraction techniques. Yet, point defect concentrations cannot be resolved below 10^−2^.

In this paper, we demonstrate how to obtain microscopic insights on the existence, nature and quantity of defects in intermetallic compounds. We combine various positron-based techniques and ab-initio calculations of point defect thermodynamics for the identification of the defect species and the quantitative determination of the defect density. This allows us to elucidate their influence on the magnetic and transport properties in the itinerant-electron magnet MnSi.

For our study we have selected MnSi as an important show-case of several aspects of new forms of electronic order. Notably, MnSi and related compounds in recent years have attracted great interest due to the formation of a regular arrangement of spin whirls forming a so-called skyrmion lattice at finite magnetic fields in a small phase pocket just below *T*_*c*_^1^,^2^,^3^,^4^,^5^,^6^,^7^,^8^,^9^,^10^. The non-trivial topological winding of these whirls gives rise to an emergent electrodynamics, in which each skyrmion carries one quantum of emergent magnetic flux^11^,^12^,^13^. The emergent electrodynamics is at the heart of exceptionally strong spin transfer torques overcoming defect related pinning. This has generated great interest to exploit skyrmions in spintronics devices. Another important facet of the magnetic properties of MnSi is the paramagnetic to helimagnetic transition, which represents the first unambiguous example of a fluctuation-induced first order transition as long predicted by Brazovskii in the context of soft matter. As for the skyrmion lattice phase the effects of tiny defect concentrations appear to be of central importance. Last but not least, high pressure studies in MnSi have revealed an extended non-Fermi liquid regime, where both the exponent and prefactor of the resistivity are essentially independent of the residual resistivity^14^,^15^,^16^. These various aspects of the importance of defects are underscored by the metallurgical properties of MnSi, which appear to be particularly amenable to the preparation of large, virtually perfect single crystals. In turn, MnSi represents a show-case par excellence in which the influence of low defect concentrations on novel electronic properties may be studied.

MnSi belongs to the cubic B20 compounds with the space group *P*2_1_3 lacking inversion symmetry. MnSi melts congruently at 1270 °C and large single crystals may be readily grown from the melt using methods such as Czochralsky, Bridgman, and inductively or optically heated float-zoning^17^. The resulting samples readily show residual resistivity ratios (RRR) between 50 and 100, where carefully annealed specimens reach values as high as 1000^14^. At ambient pressure, however, small deviations of the transition temperatures and the shapes of the corresponding thermodynamic signatures were reported for samples with different RRR^18^,^19^,^20^. The latter also seems to influence the possibility to metastably extend the skyrmion lattice pocket at large hydrostatic pressures under field-cooling^21^. However, neither the density nor the type of lattice defects in single-crystal MnSi has been addressed before. Knowledge of the defect structure in turn is key to understanding phenomena such as the very weak energy scales controlling the Brazovskii transition, the depinning of the skyrmion lattice under electrical currents^22^,^23^,^24^, the dynamics of the topological unwinding of skyrmions^25^, or the emergence of non-Fermi liquid behavior at high pressures^26^.

Given the importance of any of these topics, we decided to study the point defects in MnSi in a series of optically float-zoned single crystals by means of positron annihilation spectroscopy. In combination with calculations of the effective formation energies of point defects this allows us to determine the species and density of defects quantitatively. Measurements of the specific heat and the ac susceptibility demonstrate that the RRR is not sufficient to characterize a sample of MnSi, as specimens with the same RRR but different dominant defect species show discrepancies in the thermodynamic signatures of the magnetic phase transitions.

## Results and Discussion

**Figure 1:**
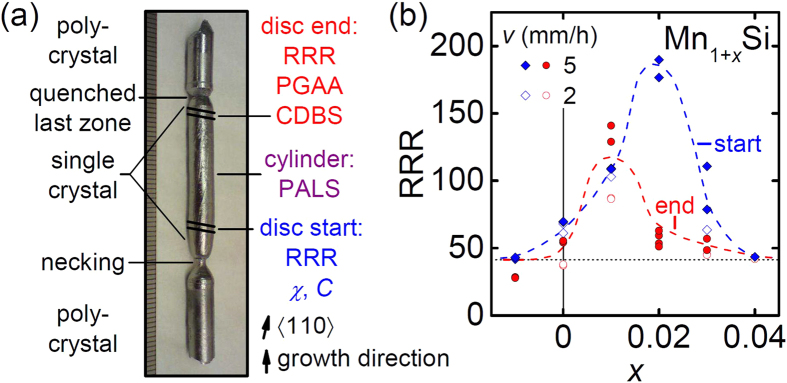
Preparation of the MnSi specimens. (**a**) Photograph of a float-zoned ingot. Single-crystal discs from the start and the end as well as the remaining cylinders were studied. (**b**) RRR as function of the initial Mn excess *x* for different growth rates *v*. Samples from the same disc exhibit very similar RRRs. Dashed lines are guides to the eye. Here and in the following figures, data acquired on discs from start, end, and the cylinders are represented respectively.

For our study ten single crystals of MnSi were grown by means of optical float-zoning^27^,^28^. We investigated the influence of two growth parameters, namely the growth rate, *v*, and the composition of the feed rods represented by the Mn excess, *x*, of the initial net weight of Mn_1+*x*_Si, −0.01 ≤ *x* ≤ 0.04. We induced this Mn excess in order to compensate evaporative losses of Mn caused by its high vapor pressure during crystal preparation. The cylinders of the single crystals (see Fig. 1(a)) were investigated by positron annihilation lifetime spectroscopy (PALS) and the end discs by coincident Doppler broadening spectroscopy (CDBS). Prompt gamma activation analysis (PGAA)^29^ results were consistent with a stoichiometric composition within the estimated error of 1.5 at.%. For all specimens we expect the same total density of impurity atoms originating from the starting elements below 5 · 10^−5^.

The RRR of all samples studied is shown in Fig. 1(b). Additionally, the values for all crystals are given in Table 1 together with the other important measured parameters introduced later. The start (blue) and end (red) of the single crystals exhibit qualitatively similar behavior, where both low and high values of *x* lead to low RRRs around 40. A maximum in the RRR, expected for a minimal total defect concentration, is obtained for a slight initial Mn excess as consistent with a compensation of the observed evaporation of Mn during crystal growth. The small systematic variations between the start and the end of the crystals may be attributed to the different temperature history during growth where the start stayed at elevated temperatures for a longer period of time leading to larger losses of Mn. In the parameter range studied, the growth rate had no significant influence on the RRR.

**Figure 2:**
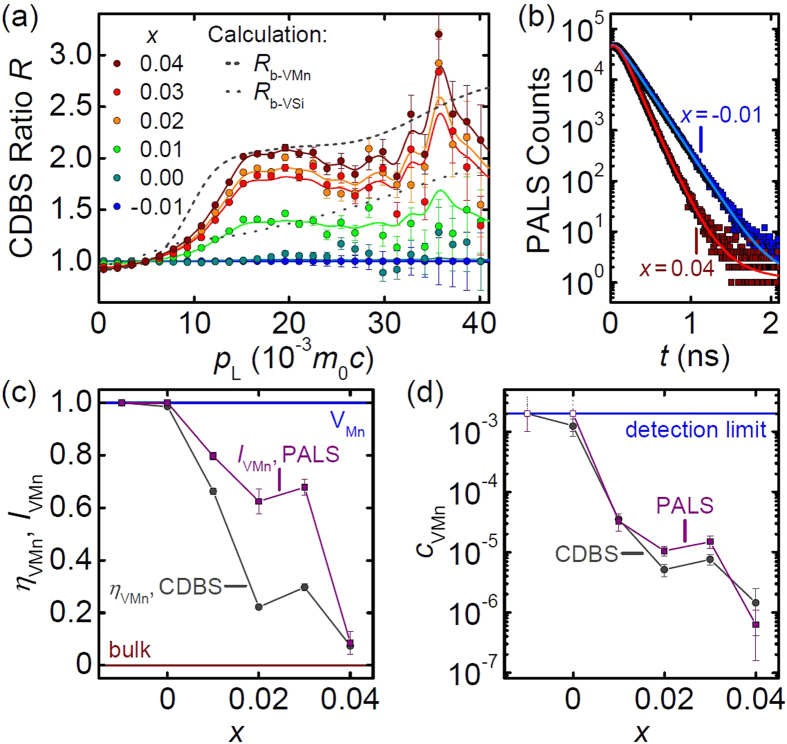
Results of the positron annihilation spectroscopy. (**a**) CDBS ratio curves *R*_x_ as function of the longitudinal momentum component *p*_L_. Solid lines represent linear superpositions of *R*_−0.01_ and *R*_0.04_, dashed lines the calculated curves *R*_b-VMn_ and *R*_b-VSi_. (**b**) PALS spectra together with fits to the data (solid lines). (**c**) Fraction *η*_VMn_ and PALS intensity *I*_VMn_ of positrons annihilating in Mn vacancies, V_Mn_, as function of *x*. (**d**) Concentration *c*_VMn_ of V_Mn_ obtained from CDBS and PALS.

The detected normalized CDB spectra are shown in Fig. 2(a) after division by the spectrum for *x* = −0.01 as so-called ratio curves, *R*_*x*_. The spectra are described very well by linear combinations of *R*_−0.01_ and *R*_0.04_ as depicted by solid lines. Here, the fitted weighting factor *f*_*x*_ is defined by *f*_−0.01_ = 0 and *f*_0.04_ = 1. The measured ratio curves *R*_*x*_ were compared to calculated ones, namely *R*_b-VMn_ and *R*_b-VSi_ of defect free bulk MnSi using either the spectra characteristic for annihilation in Mn vacancies *V*_Mn_ or Si vacancies *V*_Si_ as reference. As shown in Fig. 2(a), *R*_b-VMn_ describes well *R*_0.04_ for large momenta, *p*_L_ > 15 · 10^−3^ *m*_0_*c*, where the calculation method works with high reliability^30^,^31^,^32^,^33^. Hence, for *x* = 0.04 most positrons annihilate in the bulk. For *x* ≤ 0 the spectra hardly differ, suggesting that essentially all positrons annihilate in V_Mn_ (saturation trapping).

In order to determine the fraction, *η*_b_, of positrons annihilating in the defect-free bulk for *x* = 0.04, we compared the areas enveloped by *R*_b-Mn_ − 1 and *R*_0.04_ − 1 in the range 15.9 · 10^−3^ *m*_0_*c* < *p*_L_ < 39.5 · 10^−3^ *m*_0_*c*. We deduce that in this sample 93(5)% of the positrons annihilate in defect-free MnSi and hence *η*_b_(*x*) = 0.93 · *f*_*x*_ for the other samples. Consequently, the residual fraction *η*_VMn_(*x*) = 1 − *η*_b_(*x*) depicted in Fig. 2(c) is attributed to positrons annihilating in V_Mn_. We finally determined the concentration *c*_VMn_(*x*) = (*η*_b_(*x*)^−1^ − 1)/(*τ*_b_*μ*_VMn_), shown in Fig. 2(d), by adapting the commonly used trapping model^34^. Here, we used the calculated bulk lifetime *τ*_b_ = 111 ps and an assumed trapping coefficient *μ*_VMn_ = 5 · 10^14^ s^−1^ (Typical values of *μ* are between 10^14^ s^−1^ and 10^15^ s^−1 ^)^34^.

PALS spectra, exemplary shown in Fig. 2(b), display the same trend. For *x* ≤ 0 only one lifetime component is found with *τ*_VMn_ = 185(4) ps in excellent agreement with the calculated value of 181 ps for a positron trapped in V_Mn_. For *x* = 0.04, *τ*_VMn_ contributes only with an intensity of 8.5% to the lifetime spectrum and the dominant extracted lifetime *τ*_b_ = 119(3) ps is close to the calculated bulk value of 111 ps. Both lifetimes, *τ*_VMn_ and *τ*_b_, were fixed for analyzing the remaining crystals. Here, we determined the intensity, *I*_VMn_(*x*), shown in Fig. 2(c) arising from annihilation in V_Mn_ for the different samples using the fitted positron trapping rate *κ*_VMn_(*x*) from bulk into V_Mn_. As in CDBS, for *x* > 0 annihilation in both defect-free bulk and V_Mn_ is observed, which allowed us to calculate the concentration *c*_VMn_(*x*) = *κ*_VMn_(*x*)/*μ*_VMn_, shown in Fig. 2(d).

The values of *c*_VMn_ obtained in cylinders by PALS and discs by CDBS agree very well implying a homogeneous distribution of V_Mn_ along the cylindrical samples. For *x* ≤ 0 only a lower limit of 2 · 10^−3^ may be given for *c*_VMn_ due to saturation trapping of positrons in V_Mn_. For *x* = 0.04, the minimal vacancy concentration of *c*_VMn_ ≈ 1 · 10^−6^ establishes that an initial Mn excess efficiently suppresses the formation of V_Mn_ during crystal growth.

**Figure 3:**
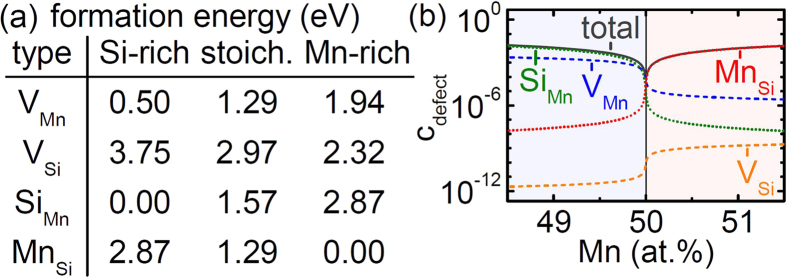
Calculations of point defect energetics. (**a**) Effective formation energies in MnSi with different composition. (**b**) Expected defect concentrations as a function of composition for MnSi around its melting temperature.

In the appropriate grand-canonical theory^35^, the internal energies of the relaxed point defect configurations yield effective formation energies for the respective defect species. Their calculated values, as summarized in Fig. 3(a), show that MnSi belongs to the class of compounds with antisite accommodation of deviations from stoichiometry (with the defect energetics described by the two dimensionless parameters *ξ* = 0.495 and *η* = −0.318)^36^. Specifically, also on the Si-rich side deviations from stoichiometry are accommodated as antisites. Still, as in this situation the thermal formation of V_Mn_ is very inexpensive, a copious equilibrium concentration of V_Mn_ is expected at the elevated temperatures during growth as illustrated in Fig. 3(b). Diffusion measurements on MnSi have not been reported yet, but data on the isostructural FeSi suggest that diffusion in B20 compounds is extremely slow^37^. Hence, large thermal vacancy concentrations will be frozen in during cooling already at high temperatures.

The results of the positron annihilation experiments in combination with the calculated formation energies of point defects allow us to interpret the RRR shown in Fig. 1(b). For starting compositions of *x* ≤ 0.01, Si antisites (Si_Mn_) and frozen-in thermal vacancies on the Mn sublattice (V_Mn_) are the dominant defect types leading to a low RRR. Around *x* ≈ 0.015, at the maximum of the RRR, a pronounced drop of *c*_VMn_ is detected which indicates a transition from Mn-deficient to stoichiometric crystals with a minimal total concentration of defects. The shift with respect to *x* = 0 is attributed to the evaporation of Mn during growth. For *x* ≥ 0.02, *c*_VMn_ further decreases as observed by CDBS and PALS. Inferred from the effective formation energies, the low RRR in this Mn-rich regime is mainly attributed to Mn antisites on the Si sublattice (Mn_Si_). It is noteworthy that contributions from vacancies on the Si sublattice (V_Si_) are neither detected experimentally nor expected from calculations. Contributions to the RRR arising from scattering on impurity atoms are assumed to be similar for all samples studied.

**Figure 4:**
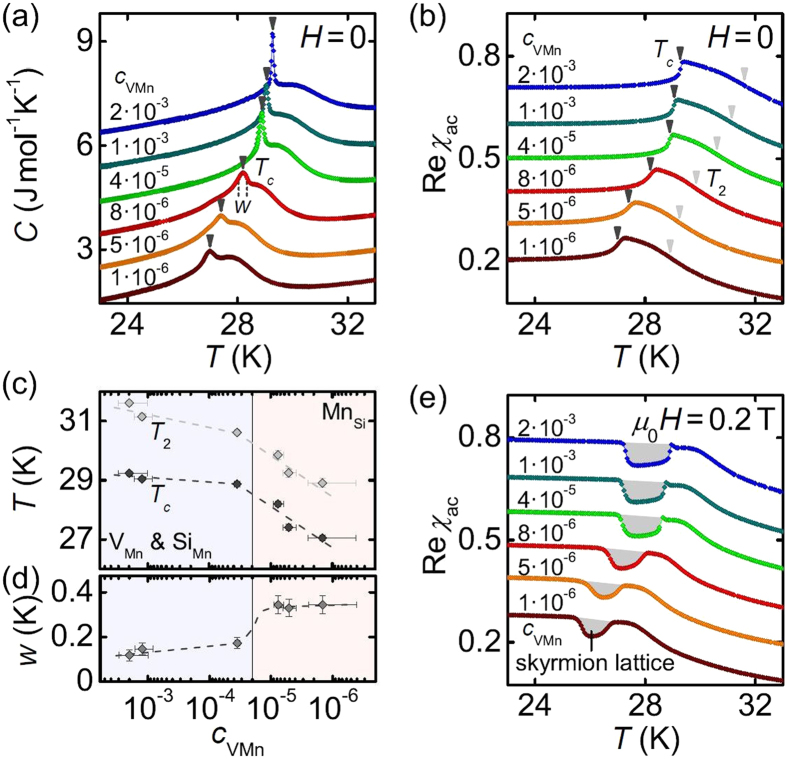
Magnetic properties of MnSi with different defect concentrations as determined by positron spectroscopy. (**a**,**b**) Temperature dependence of the specific heat, *C*, and the real part of the ac susceptibility, Re*χ*_ac_, for samples with different concentrations of Mn vacancies, *c*_VMn_. (**c**) Transition temperatures *T*_*c*_ and *T*_2_ as function of *c*_VMn_. We distinguish two regimes with different dominant defect species. (**d**) Temperature width, *w*, of the transition at *T*_*c*_ as function of *c*_VMn_. (**e**) Susceptibility in finite fields applied along 〈100〉 with a minimum attributed to a skyrmion lattice state (gray shading). Data have been offset for clarity.

Figure 4 finally addresses the magnetic properties of different samples at temperatures around the helimagnetic phase transition. The specific heat as a function of temperature shown in Fig. 4(a) is qualitatively very similar for all samples. With decreasing temperature a broad maximum is associated with the fluctuation-disordered (FD) regime, while a sharp peak marks the first-order transition at *T*_*c*_. These findings are corroborated by the magnetic ac susceptibility, see Fig. 4(b), where a point of inflection at *T*_2_ defines the crossover from the paramagnet at high temperatures to the FD regime^38^,^39^,^40^.

As shown in Fig. 4(c), with decreasing concentration of Mn vacancies, *c*_VMn_, both *T*_*c*_ and *T*_2_ monotonically decrease. The temperature range of the FD regime, *T*_2_ − *T*_*c*_, remains essentially unchanged. In addition, the transition at *T*_*c*_ broadens as indicated by the full temperature width at half maximum, *w*, of the specific heat anomaly depicted in Fig. 4(d). A prominent change of slope around *c*_VMn_=2 · 10^−5^ divides our samples in two groups with either high or low concentration of Mn vacancies. As established above, Si-rich samples are dominated by Mn vacancies and Si antisites (high *c*_VMn_, blue shading) while Mn-rich specimens are dominated by Mn antisites (low *c*_VMn_, red shading). Taken together, our findings imply that in particular Mn antisite disorder leads to a suppression of the transition temperatures accompanied by a smearing of the first-order transition at *T*_*c*_. Still, as depicted in Fig. 4(e), all samples investigated show a plateau of reduced susceptibility just below *T*_*c*_ in an applied field of 0.2 T. This signature is characteristic of the skyrmion lattice state.

The shift of *T*_*c*_ and *T*_2_ suggests that Mn antisites directly modify the electronic structure and in turn potentially all magnetic interactions. Apparently, ferromagnetic ordering is weakened by this kind of defects. Understanding the microscopic mechanisms, however, will require ab-initio calculations (see e.g. ref. 41). We finally note that in MnSi defect concentrations of 10^−3^ and 10^−6^ translate to mean defect distances of 5 and 50 lattice constants, respectively, as compared to the helix wavelength of 180 Å corresponding to 40 lattice constants. Bearing in mind the very large magnetic correlation length of ~10^4^ Å in bulk MnSi, defect-related pinning of magnetic textures is expected mainly in form of (weak) collective pinning^42^.

## Conclusion

We have combined measurements of the magnetic bulk properties, RRR, positron annihilation spectroscopy, and calculations of effective defect formation energies in order to identify the species of point defects and their influence on magnetic and transport properties in a series of single crystals of MnSi. Using this approach we gained a detailed picture of characteristic lattice defects. Beside antisites on both sublattices, vacancies on the Mn sublattice are immanent in the system, where the dominant type of defects can be tuned by the initial Mn content. The magnetic properties of MnSi are qualitatively extremely robust to the defect concentration including the formation of the skyrmion lattice state. However, Mn antisites shift the transition temperatures to lower values and broaden the first-order transitions. Similar consequences may also be expected for the emergent phenomena of the skyrmion lattice. These new findings can serve as benchmark for microscopic theories on the complex magnetic behavior of MnSi. Hence, albeit the RRR may essentially reflect the absolute density of defects, the knowledge on the type of defects is key when characterizing single crystals of MnSi.

## Methods

### Measured Parameters

All measured quantities are summarized in Table 1. The principles of the applied experimental techniques and the accompanying theoretical calculations are explained in the following.

### Crystal Preparation and Characterization

All crystal growth was carried out after evacuating the furnaces to about 10^−7^ mbar and filling them with 1.5 bar of 6 N Ar treated with a point-of-use gas purifier^43^. First, high-purity elements (precast 4 N Mn and 6 N Si) were alloyed in an inductively heated furnace and cooled down in less than 5 min. In order to ensure compositional homogeneity, the resulting ingots were flipped and remolten three times before being cast to polycrystalline feed rods. Two rods of identical starting composition were optically float-zoned at a rate of 2 mm/h or 5 mm/h, respectively, while feed and seed rod were counter-rotating at 6 rpm. A necking during the first millimeters of growth promoted grain selection. Despite the argon atmosphere, the high vapor pressure of Mn leads to small losses. All growth attempts produced single crystals of 6 mm diameter and 10–30 mm length with no preferred direction of growth as determined by X-ray Laue diffraction. From the start and the end of the single crystals, see Fig. 1(a), we cut discs of 1 mm thickness perpendicular to 〈110〉 using a wire saw. From each disc we prepared two platelets of ~5 × 1 × 0.2 mm^3^ with their long edge along 〈100〉.

The RRR of these samples was measured in a 4-terminal configuration using a bespoke dipstick in a liquid helium dewar and a standard lock-in technique. Specific heat, *C*, and magnetic ac susceptibility, Re*χ*_ac_, were measured in a QD-PPMS on cubes of 1 mm edge length prepared from the start discs. For the specific heat we used a quasi-adiabatic heat pulse method where pulses had a size of 30% of the sample temperature^40^. The susceptibility was measured at an excitation frequency of 911 Hz with an amplitude of 1 mT.

### Positron Annihilation Spectroscopy

In general, open-volume defects such as vacancies can trap positrons prior to annihilation leading to longer lifetimes than in the bulk due to the locally reduced electron density. PALS allows to detect the resulting annihilation rates and attribute them to defect-free bulk or vacancies^44^. In CDBS^45^,^46^ measuring the energy of both annihilation *γ*-quanta yields the Doppler shift 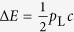 caused by the longitudinal momentum component, *p*_L_, of the annihilating pair. In vacancies Δ*E* is smaller than in bulk due to the lower overlap of the localized positron wave function with high-momentum core electrons. Furthermore, due to the intrinsically low background, CDBS allows to examine the chemical surrounding of vacancies^45^.

CDBS was performed at the high-intensity positron beamline NEPOMUC at MLZ^47^,^48^ with an incident positron energy of 25 keV corresponding to a mean implantation depth of 1.3 *μ*m in MnSi. Beforehand, we confirmed that the bulk of the samples was probed by variation of the beam energy. Complementary PALS was carried out at the GIPS facility by generating positrons in the bulk of the cylindrical samples from a high-energy pulsed *γ*-beam^49^. The lifetime spectra were detected in a coincidence setup and analyzed by least-square fits.

In addition, we calculated CDB spectra and positron lifetimes in MnSi for annihilation in bulk as well as in vacancies V_Mn_ and V_Si_ on the Mn and Si sublattices. We used the MIKA Doppler program^50^, which computes the positron wave function with a two-component density functional theory in the limit of a vanishing positron density^51^ and describes the electron density based on an atomic superposition method^52^. After convolution of the calculated spectra with the experimental resolution, the bulk CDB ratio curves *R*_b-Mn_ and *R*_b-VSi_ were evaluated using spectra for V_Mn_ and V_Si_ as reference.

### Ab-initio Calculations on Point Defects

Point defect energetics were calculated via density-functional theory (PBE-generalized gradient approximation^53^) as implemented in the ABINIT code using the projector-augmented wave framework^54^. We used a plane-wave cut-off of 30 Ha and a shifted fcc *k*-point grid corresponding to 32 points in the Brillouin zone of the ideal B20 simple cubic unit cell. For the defect-free system, the lattice constant yielded *a* = 4.499 Å, compared to the room temperature experimental value of 4.560 Å^55^. For the point defect calculations, a bcc arrangement with four eight-atom B20 cells per supercell was chosen, with one of these 32 atoms either removed or substituted by the other constituent element. Atomic positions were relaxed under fixed supercell dimensions. As the relevant temperature range for point defect creation and removal is far above the magnetic ordering temperature, no spin polarization was allowed.

## Additional Information

**How to cite this article**: Reiner, M. *et al*. Positron spectroscopy of point defects in the skyrmion-lattice compound MnSi. *Sci. Rep*. **6**, 29109; doi: 10.1038/srep29109 (2016).

## Figures and Tables

**Table 1:** Physical parameters of the investigated MnSi single crystals.

*x*	RRR		PAS Observables		*c*_VMn_		Magnetic Transition		
	start	end	*η*_VMn_	*I*_VMn_	discs	cylinders	*T*_c_(K)	*T*_2_(K)	*w*(K)
−0.01	42.5	28.1	1.00	1.00	>2 · 10^−3^	>2 · 10^−3^	29.2	31.6	0.12
0.00	69.6	54.8	0.99	1.00	1.2 · 10^−3^	>2 · 10^−3^	29.0	31.1	0.15
0.01	109	135	0.66	0.80	3.5 · 10^−5^	3.3 · 10^−5^	28.9	30.6	0.17
0.02	183	56.7	0.22	0.62	5.1 · 10^−6^	1.0 · 10^−5^	27.4	29.3	0.33
0.03	94.7	52.7	0.30	0.68	7.6 · 10^−6^	1.5 · 10^−5^	28.2	29.9	0.34
0.04	43.5	41.9	0.074	0.044	1.4 · 10^−6^	6.3 · 10^−7^	27.1	28.9	0.34
Initial Mn excess *x* of sample preparation, RRR at start and end of crystals, PAS observables *η*_VMn_ and *I*_VMn_ determined from CDBS and PALS, respectively, (with values of 1.00 displaying saturation trapping of positrons in V_Mn_), vacancy concentrations *c*_VMn_ in discs and cylinders, transition temperatures *T*_*c*_ and *T*_2_ as well as the temperature width *w* of *T*_*c*_.									
